# Evaluation of Techniques for Measuring Microbial Hazards in Bathing Waters: A Comparative Study

**DOI:** 10.1371/journal.pone.0155848

**Published:** 2016-05-23

**Authors:** Christelle Schang, Rebekah Henry, Peter A. Kolotelo, Toby Prosser, Nick Crosbie, Trish Grant, Darren Cottam, Peter O’Brien, Scott Coutts, Ana Deletic, David T. McCarthy

**Affiliations:** 1 Environmental and Public Health Microbiology Laboratory (EPHM Lab), Monash University, Clayton, Victoria, Australia; 2 Melbourne Water, Docklands, Victoria, Australia; 3 Environment Protection Authority Victoria, Melbourne, Victoria, Australia; 4 Mornington Peninsula Shire, Rosebud, Victoria, Australia; 5 Micromon, Monash University, Clayton, Victoria, Australia; University of Brighton, UNITED KINGDOM

## Abstract

Recreational water quality is commonly monitored by means of culture based faecal indicator organism (FIOs) assays. However, these methods are costly and time-consuming; a serious disadvantage when combined with issues such as non-specificity and user bias. New culture and molecular methods have been developed to counter these drawbacks. This study compared industry-standard IDEXX methods (Colilert and Enterolert) with three alternative approaches: 1) TECTA™ system for *E*. *coli* and enterococci; 2) US EPA’s 1611 method (qPCR based enterococci enumeration); and 3) Next Generation Sequencing (NGS). Water samples (233) were collected from riverine, estuarine and marine environments over the 2014–2015 summer period and analysed by the four methods. The results demonstrated that *E*. *coli* and coliform densities, inferred by the IDEXX system, correlated strongly with the TECTA™ system. The TECTA™ system had further advantages in faster turnaround times (~12 hrs from sample receipt to result compared to 24 hrs); no staff time required for interpretation and less user bias (results are automatically calculated, compared to subjective colorimetric decisions). The US EPA Method 1611 qPCR method also showed significant correlation with the IDEXX enterococci method; but had significant disadvantages such as highly technical analysis and higher operational costs (330% of IDEXX). The NGS method demonstrated statistically significant correlations between IDEXX and the proportions of sequences belonging to FIOs, *Enterobacteriaceae*, and *Enterococcaceae*. While costs (3,000% of IDEXX) and analysis time (300% of IDEXX) were found to be significant drawbacks of NGS, rapid technological advances in this field will soon see it widely adopted.

## Introduction

Bays, estuaries, and rivers provide ecological, economical, and recreational values to the community [1, 2]; but they are under constant and increasing pressure from urbanisation, population growth, and a changing climate [3]. Faecal contamination remains, in coastal and inland regions around the world, the primary cause of closure for recreational use [4]. Melbourne’s Yarra River and Port Phillip Bay are no exception, very often under scrutiny for their pathogen levels.

Faecal indicator bacteria are commonly used to estimate human health risks in recreational waters internationally–including enterococci for marine waters and *E*. *coli* for fresh waters [1, 2, 5]. Techniques based on defined substrate cultures, such as the IDEXX methods [6], are commonly used to quantify indicator levels because of their relative ease of use, low cost, and epidemiological evidence that links such levels to human illness [7]. They have therefore become the effective industry standard in Melbourne, Australia. But these techniques have at least four drawbacks: (a) they take at least 18 hours to complete (meaning slower reporting to community about risks, or indeed significant divergences between reported and current risks [8]); (b) they require lab personnel to analyse and report results the following day (so weekend staffing issues often make Friday samples problematic); (c) they can introduce user bias (for example colorimetric systems, by relying on visual comparisons with templates, are prey to systematic or arbitrary error); and (d) they are known to have associated specificity issues [9].

New techniques are being developed to address such shortfalls. For example, the new TECTA™ pathogen detection system uses an optical fluorescence sensor, directly coupled to a bacteria culture test, to estimate cell densities in water samples for *E*. *coli*, total coliforms, and (in the near future) enterococci [10, 11]. Fluorescence is used to measure enzyme activity of target organisms, and is automatically converted to concentrations without appeal to subjective visual interpretation –in a fraction of the time required by traditional cell-culture techniques.

Methods have also been developed that directly measure DNA, RNA, or surface immunological properties rather than bacterial growth and metabolic activity. These enable faster, more specific and more accurate measurements of bacteria in water [12, 13]. *Quantitative polymerase chain reaction* (qPCR) techniques, for *Escherichia coli* or *Enterococcus* spp., have shown promising results and significant correlation with traditional culture-based methods [14]; but these have not been widely tested in oceanic climates such as Melbourne’s. Although these molecular techniques rarely yield information about the viability of cells, links have been established between the concentrations they measure and human health outcomes [15].

Next Generation Sequencing (NGS) can also furnish information about the levels of bacteria communities in water samples [16]. While costs and processing times are assumed to be higher for NGS than for the other methods, advances in technology and computing power will soon drive these costs to comparable levels. Furthermore, the significant gain in having entire bacterial community profiles (not just one or two indicators) enables multiple lines of evidence for estimating risks to human health [17], and for identifying any complex mixture of pollution sources in contaminated recreational waters using tools such as microbial source tracking [18–20]. To our knowledge however, there has been no comparative study of results from NGS and the more traditional techniques for proportions of particular families or species of bacteria.

In this paper, the performance of the traditional IDEXX defined-substrate methods for *E*. *coli* and enterococci are compared with three novel methods: 1) TECTA™ (culture-based *E*. *coli* and enterococci enumeration), 2) US EPA’s 1611 method (qPCR-based enterococci enumeration), and 3) NGS. Methods 1 and 2 promise faster reporting times, eliminate operator input on the following day, and reduce user bias; method 3 promises to deliver a more detailed assessment of faecal pollution in a given sample. Our study focused on Melbourne’s recreational waters, with samples collected over the 2014–2015 swimming season for analysis by all four methods. We also compared estimated cost, operator time, and time to reporting for the methods.

## Materials and Methods

For this study, field permits were granted by Melbourne Water, Environmental Protection Authority Victoria and Mornington Peninsula Shire.

### Sample collection

**Fig 1:**
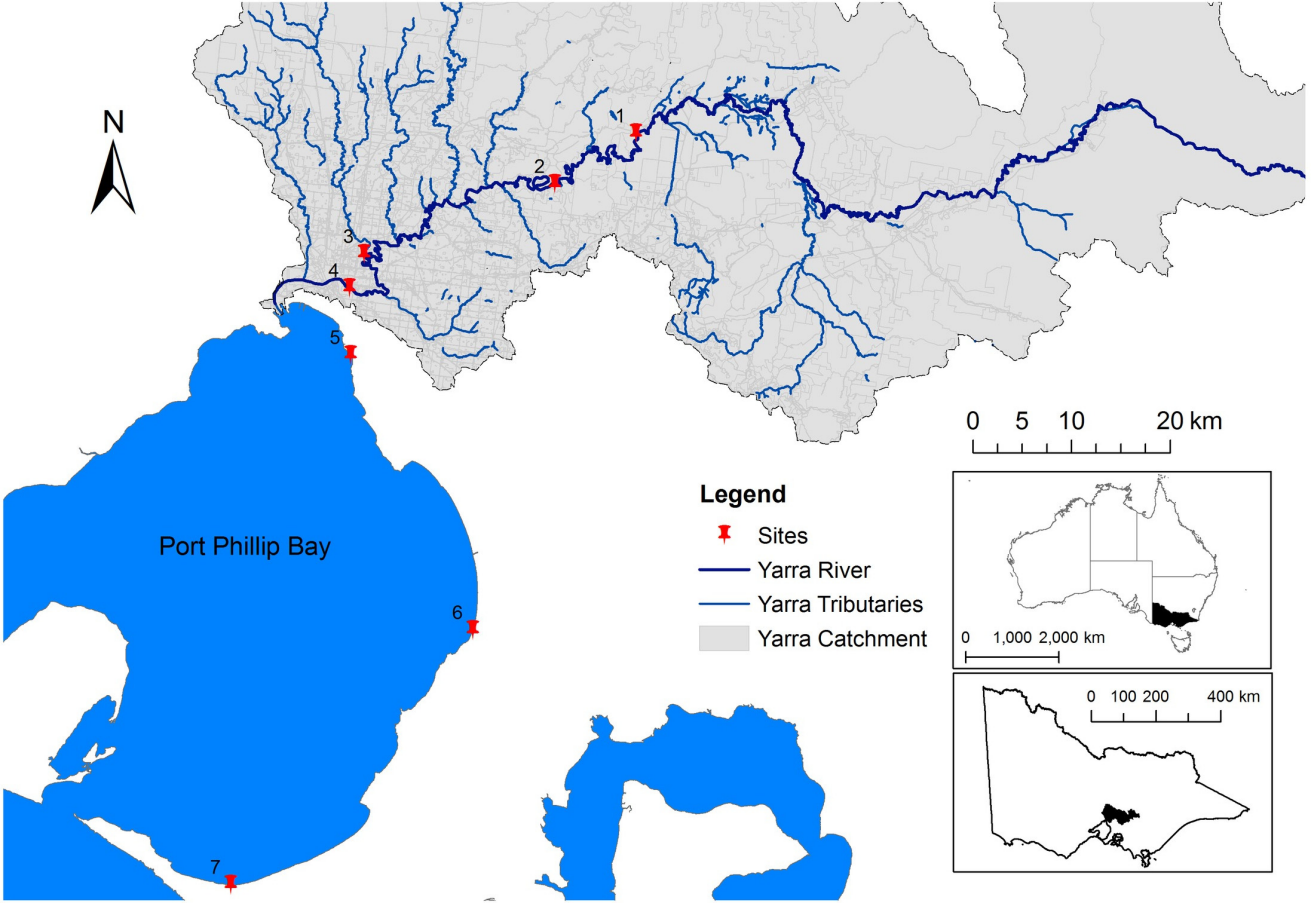
Site locations along the Yarra River, the Yarra Estuary, and Port Phillip Bay in Melbourne, Australia. Site details provided in Table 1.

233 water samples were collected three to four times a week, between December 2014 and March 2015, from the following: three beach sites along the east coast of Port Phillip Bay (ELW, FRA, and RYE), one site in the estuarine part of the Yarra River (MOR), and three fresh water river sites (DFS, WAR, and YER). This period was chosen to coincide with the southern hemisphere beach season and the EPA Victoria Beach Report season. Table 1 summarises the site location and monitoring periods, and the locations are mapped in Fig 1.

**Table 1:** Site location and characteristics.

Site	Ref.	Site name	Location	Monitoring period	GPS location
1	YER	Yering	Yarra River upstream	Jan–Mar 2015	-37.691698; 145.317018
2	WAR	Warrandyte	Yarra River upstream	Jan–Mar 2015	-37.736517; 145.222720
3	DFS	Dights Falls	Yarra River upstream	Dec 2014 –Mar 2015	-37.796988; 145.001416
				2–5 Mar 2015^#^	-37.796878; 145.000175
4	MOR	Morell Bridge	Yarra River estuary	Dec 2014 –Mar 2015	-37.827752; 144.983937
5	ELW	Elwood Beach	Port Phillip Bay	Dec 2014 –Mar 2015	-37.889094; 144.983612
6	FRA	Frankston Beach	Port Phillip Bay	Dec 2014 –Mar 2015	-38.142595; 145.118282
7	RYE	Rye Beach	Port Phillip Bay	Dec 2014 –Mar 2015	-38.370196; 144.831464

^#^ Because of construction work at Dights Falls, three samples were taken at a slightly different location between 2 and 5 March.

### Sample analysis

Three different methods were used to analyse samples for common faecal indicator organisms (FIOs) *E*. *coli* and enterococci: IDEXX methods [6], TECTA™ [10, 11], and US EPA Method 1611 qPCR [21]. As per the State Environment Protection Policy (SEPP) and the Australian Guidelines for Managing Risks in Recreational Waters [1, 2, 22], upstream and estuarine water samples were tested for *E*. *coli* while marine samples were tested for enterococci (Table 2). Samples were also analysed for bacterial communities using Next Generation Sequencing (NGS) approach; due to costs, only 62 samples were carefully selected for analysis (based on getting good distribution across freshwater and marine water sites, wet and dry weather periods and high/low levels of indicator organisms).

**Table 2:** Analyses performed on samples.

Site	IDEXX	IDEXX	Total coliform and *E*.* coli*	Enterococci	Enterococci	Next-Generation
	Colilert	Enterolert	TECTA™	TECTA™	US EPA Method 1611	Sequencing (NGS)
YER	X		X			X
WAR	X		X			X
DFS	X	X^1^	X			X
MOR	X	X^1^	X			X
ELW		X		X	X	X
FRA		X		X	X	X
RYE		X		X	X	X

^1^ Samples were also tested using IDEXX’s Enterolert for these samples to allow for comparison with the NGS method from 05/02/2015 until the end of the sampling period.

#### IDEXX analysis

All samples were analysed according to guidelines from the manufacturer (IDEXX), including method blanks and spikes. Before the addition of the Colilert or Enterolert reagents, all samples were diluted 1:10. Quanti-trays were sealed and then incubated for 24 hours at 35±1°C (*E*. *coli*) or 41±1°C (enterococci). Trays were then compared to comparators, and positive wells were counted and transformed to determine *most probable numbers* (MPNs) using the provided IDEXX MPN charts. Duplicate samples collected were also sent to a NATA-approved laboratory for enterococci and *E*. *coli* analyses.

#### TECTA™ analysis

All samples were analysed according to the manufacturer’s guidelines (ENDETEC™), including method blanks and spikes. 10 mL of samples were added into the TECTA™ cartridges containing defined-substrate growth media –with glucuronidase and galactosidase enzymes for the detection of *E*. *coli* and total coliform respectively, or glucosidase enzyme for the detection of enterococci. For the detection of *E*. *coli*, the cartridges were then filled with 30 mL of filtered and sterilised water. To test for presence of enterococci, 20 ml of ENA solution (Veolia) was added to the sample together with 70 mL of filtered and sterilised water. Samples were gently mixed until reagents were fully dissolved. The cartridges were then placed into the TECTA™ benchtop instrument. *E*. *coli* and total coliform samples were incubated at a constant 35°C, and the enterococci samples at 41°C. The fluorescence spectrum was then continuously monitored through the optical fibre coupled to the spectrometer mounted in the instrument. Fluorescence measurements indicating time of detection of enzyme activity were automatically translated into a concentration of colony forming unit per 100mL (cfu/100 mL), and automatically sent by email to the operators along with a report of any fault in the instrument during incubation.

#### EPA 1611 analysis

The procedures outlined in US EPA 1611 were followed [21]. The following is a brief overview of the techniques (for full details see [21]).

*DNA standard*, *calibrators*, *spiked matrix*, *and negative control samples*. DNA standard and calibrator samples were prepared for extraction and analysed in advance of the study, then stored at -80°C. Each week, triplicate calibrators were extracted and analysed with each batch of water samples. For the preparation of calibrators, 10^5^ cells of laboratory-grown *Enterococcus faecalis* (strain ATCC#29212) were suspended in 590 μL AE buffer (Qiagen, Valencia, CA) containing 0.2 μg/mL salmon DNA (#D-1626, Sigma, St Louis, MO) and transferred to extraction tubes containing glass beads and fitted with polycarbonate filters (Millipore HTTP04700; 0.45 μm pore size, 47 mm diameter). Matrix-spiked water and spiked phosphate-buffered saline (PBS) samples were tested each week alongside other water samples by adding an estimated 550 cfu/100 mL of freshly grown *E*. *faecalis* to each sample. The grown culture was then plated on brain and heart infusion agar plates to determine the true concentration of the spiking solution. Negative control samples were prepared using PBS and prepared in the same manner as the water samples. All control samples were extracted and analysed on the day of preparation.

*DNA extraction for enterococci Qpcr*. 100 mL of the water sample was filtered through polycarbonate membrane filters (Millipore, HTTP04700; as above) and the filtration unit was post-rinsed with an additional 20 mL PBS [21]. Filters were taken from the filtration unit, carefully folded, and inserted in 2 mL extraction tubes containing acid-washed glass beads of diameter 212−300 μm (GeneRite, NJ, US, S0205-50). Extraction tubes were stored at -80°C until the day of extraction. Once a week, samples were thawed and 590 μL of 0.2 μg/mL salmon DNA in AE buffer (Qiagen, Valencia, CA) were added to each extraction tube. Cells were then re-suspended from the filters and lysed in a bead beater (Fast-Prep®24, MP Biomedicals) for 60 s at speed 6.0 m/s before being centrifuged for 1 min at 12,000 x g to pellet the glass beads and debris. The supernatants were transferred to sterile low-retention 1.7 mL microcentrifuge tubes and centrifuged a second time for 5 min. These supernatants (DNA extracts) were then diluted 5-fold using AE buffer, and analysed by qPCR.

*qPCR analysis*. Amplification assays were performed in a BIORAD CFX96 thermal cycler. Lyophilised (bead) assays–TaqMan® Enterococcus spp. (#4485045, LifeTechnologies) and TaqMan® Oncorhynchus keta (#4485045, Life Technologies) –were used for this monitoring period. 30 μL of 5-fold diluted water sample, calibrators, and controls were added to each PCR well containing the reagent; this was done in duplicate, unless otherwise specified. Samples were then gently vortexed and centrifuged before starting the thermal cycling. Thermal cycling conditions for all reactions were 45 cycles of 15 s at 95°C and 2 min at 60°C, after an initial incubation at 50°C for 2 min and 95°C for 10 min, as described in Method 1611 [21]. Data were analysed at a threshold of 1000 RFU on the thermal cycler. Cycle threshold (Cq) values were then exported to Excel for further analysis. Ongoing recovery efficiencies, matrix spike recoveries, calibrator data, and controls were all conducted in accordance with Method 1611 [21].

#### NGS analysis

The procedure for NGS analysis is described below.

*Sample filtration*. Up to 1 L of each water sample was filtered each day and collected on a maximum of 5 x 0.22 μm mixed ester cellulose filters (Millipore, GSWG047S6). Once a sample was filtered, the edges of the filtration unit were rinsed with 20 mL of water that was free of DNA and RNA. Filters were placed in a γ-sterile container and stored at -20°C until DNA extraction.

*Sample extraction*. Filters were frozen for a minimum of 2 hours at -80°C and crushed into coarse-soil-sized particles using a tool sterilised with a Bunsen burner. Total genomic DNA was isolated using PowerSoil Max DNA isolation kits (MoBio, #12988–10) following manufacturer’s instructions but with the following modifications: A shaking waterbath set at 65°C was used to lyse cells present in the samples after addition of buffer C1 for 45 min; the column membranes were incubated at room temperature for 10 min before elution of the membrane; DNA was eluted in a final volume of 1.5 mL of buffer C6, then stored at -20°C until sequencing.

*Amplification and sequencing*. The V3-4 region of the bacterial rRNA gene was amplified for each sample in triplicate using 50 μL PCR reactions: 5 μL of genomic DNA (except negative controls, where ultrapure water was substituted for the DNA), 1 x PCR buffer (Roche), 0.3 μL of Taq polymerase (Roche), 0.1 μM of forward primer (5’-TCGTCGGCAGCGTCAGATGTGTATAAGAGACAGCCTACGGGNGGCWGCAG) and of reverse primer (5’-GTCTCGTGGGCTCGGAGATGTGTATAAGAGACAGGACTACHVGGGTATCTAATCC), and the remaining volume of ultrapure water. Reactions were subjected to an initial denaturation at 98°C followed by 25 cycles of denaturation at 98°C, annealing at 55°C and extension at 72°C, each for 30 seconds. A final extension was carried out for 5 min at 72°C. The resulting PCR products were purified using 0.6 volumes of Ampure XP according to manufacturer’s instructions. 5 μL of purified PCR product was subjected to secondary PCR amplification to facilitate the addition of Illumina-compatible sequencing adapters and unique per-sample indexes; 50 μL PCR reactions were constructed to contain 5 μL each of the forward and reverse primers from the Nextera XT DNA Sample Preparation Kit (Illumina, San Diego, California, U.S.A.), 25 μL of 2 x KAPA HiFi HotStart ReadyMix (KAPA Biosystems, Wilmington Massachusetts, U.S.A.), and the remaining volume of ultrapure water. The DNA from the triplicate reactions was pooled and purified using 0.6 V of Ampure XP (Beckman Coulter, Brea California, U.S.A.), to manufacturer’s instructions. The resulting amplicon mix for each sample was quantified using Invitrogen Qubit and QuantIT reagents (Invitrogen, Grand Island New York, U.S.A.), normalised, pooled, and sequenced using a MiSeq V3 600c Reagent Kit (Illumina, San Diego California, U.S.A.) according to manufacturer’s instructions.

*Quality filtering and OTU picking*. Sequencing data were demultiplexed on the sequencing instrument using MiSeq Reporter V2.4.60 and quality-trimmed and adapter-filtered using Trimmomatic [23]. Reads were filtered to remove sequencing adapters, and trimmed to remove any terminal stretches of bases at or below Q30. After processing, any reads shorter than 180 bases were discarded. Trimmed and filtered read pairs were assembled on a pre-cluster basis to produce single reads using PEAR [24], allowing a minimum overlap of 20 bases. The assembled reads were analysed to identify operational taxonomic units (OTUs) using the QIIME 1.8.0 open-reference OTU picking workflow (clustered at >97% similarity) with UCLUST for *de novo* OTU picking, and the GreenGenes 13_8 release (clustered at 97% similarity) for the reference and for taxonomic identity assignment (full QIIME scripts available in S1 Appendix). The complete minimal data set for all samples is available on the Short Read Archive (http://www.ncbi.nlm.nih.gov/sra/; project reference PRJNA309092) to allow replication of the NGS findings of this study.

### Data analysis

For all data, results below the detection limit were taken as equal to half the detection limit, and when results were above the upper detection limit, concentrations were taken as equal to the upper detection limit. However, it is important to note that *E*. *coli* and enterococci concentrations were always below the upper detection limit (20820 MPN/100 mL); that is, only total coliforms were ever above the upper detection limit. Data from the qPCR method were standardised by correcting the comparative cell concentration according to the average OPR sample recovery rate (316%).

#### Comparisons to guidelines and policy

The data collected in this report were examined against relevant regulations and guidelines. First, comparisons were made with the NHMRC Guidelines for Managing Risks in Recreational Water Quality [22], which are in part based on the Australian and New Zealand guidelines for fresh and marine water quality [5]. The guidelines set out four risk categories, A to D, dependent on the 95th-percentile enterococci concentration. Category A (<40 enterococci/100 mL) corresponds to a low risk, while at the other extreme category D (>500 enterococci/100 mL) represents a significant risk of illness transmissions. We calculated the 95th-percentile enterococci data for each marine and estuarine site using the IDEXX Enterolert method, and set these against the guidelines to derive a health-risk classification.

Comparisons were also made with the SEPP: Waters of Victoria [1, 2], for long-term water quality assessment. SEPP sets specific objectives for different water systems. For the Yarra River catchment (i.e YER, WAR, DFS, MOR) the policy specifies two indicator levels; one for primary-contact recreation (median *E*. *coli* <150 MPN/100 mL) and another for secondary-contact recreation (median *E*. *coli* <1000 MPN/100 mL). For Port Phillip Bay (i.e. ELW, FRA, RYE), the policy also specifies two long term objectives for bacteriological indicators (one using geometric mean <200 MPN/100 mL and one using the 80th-percentile <400 MPN/100 mL). We directly compared our collected *E*. *coli* and enterococci data (obtained from IDEXX methods) to these SEPP numbers.

#### Inter-method comparisons

As neither the raw data and the log transformed data were normally distributed (Shapiro-Wilk and Kolmogorov-Smirnov; p<0.05), a non-parametric statistical approach (Spearman Rank Correlation Coefficient; [25]) was used to assess the agreement between: a) IDEXX and TECTA™ methods for *E*. *coli*, enterococci and total coliforms, b) IDEXX and Method 1611 qPCR methods for enterococci and c) IDEXX and NGS methods.

#### Influence of rainfall

Rainfall data collected in 6 minute intervals from rain gauges close to the sampling sites were used to assess the influence of rainfall on *E*. *coli* and enterococci concentrations during the monitoring period (Table 3). In some cases rainfall measurements were averaged across gauges, to take into account the upstream catchment contribution. Rainfall for the previous 24, 48, and 72 hours was calculated for each of the samples collected from December 2014 to March 2015. The antecedent dry period was also estimated from these data, by calculating the number of days with rainfall greater than 1 mm in the antecedent 24 hours.

**Table 3:** Rain-gauge combinations used for rainfall analysis at each sampling site.

Sampling site	Rain gauges	Gauge reference numbers(Source: Melbourne Water)
Yering	Yering	229247B
Warrandyte	Warrandyte, Yering	229200B, 229247B
Dights Falls	Fairfield, Warrandyte, Yering	229143A, 229200B, 229247B
Morell	Burnley, Fairfield, Warrandyte, Yering	229621A, 229143A, 229200B, 229247B
Elwood	Elsternwick, St Kilda	229660A, 229670A
Frankston	Frankston North	228378A
Rye	Seawing National park	586202

#### NGS data analysis

For each sample analysed by NGS, community profiles were obtained showing a list of unique OTUs and the proportion of the sample’s sequences assigned to each. The concentrations of *E*. *coli* and enterococci from the methods outlined above were compared against the proportion of each sample’s sequences belonging to: 1) the family Enterobacteriaceae (to which *E*. *coli* belongs), 2) the family Enterococcaceae (to which enterococci belong), 3) *Escherichia*, 4) *Enterococcus*, and 5) our own defined FIO list. Our own list was founded on the evidence of a number of papers [26–38], and in particular on whether they have already been used in water quality guidelines [1, 2, 5, 22, 39]. It includes *Aeromonas*, *Bacteroides*, *Bifidobacterium*, *Citrobacter*, *Enterobacter*, *Eubacterium*, *Methanobrevibacter*, *Campylobacter*, *C*. *coli*, *C*. *jejuni*, *Enterococcus*, *Escherichia*, *E*. *coli*, *Klebsiella*, *Moraxella*, *Proteus*, *Salmonella*, *Clostridium perfringens*, and *Pseudomonas aeruginosa*. To explore the potential of NGS further, community profiles were searched for a range of organisms causing disease in humans–limited to established faecal-derived bacteria [26–38] and some pathogens that are transmitted zoonotically (that is, to humans from other animals) [39, 40]. The following genus were included in each search (detailed list of species included in each search is provided in S1 Appendix): *Acinobacter*, *Aeromonas*, *Anaplasma*, *Bacillus*, *Bartonella*, *Borrelia*, *Brucella*, *Burholderia*, *Campylobacter*, *Candidatus*, *Chlamydia*, *Chlamudophila*, *Clostridium*, *Corynebacterium*, *Coxiella*, *Ehrlichia*, *Elizabethkingia*, *Francisella*, *Haemophilus*, *Helicobacter*, *Klebsiella*, *Legionella*, *Listeria*, *Mycobacterium*, *Mycoplasma*, *Neisserua*, *Neorickettsia*, *Orientia*, *Parachlamydia*, *Proteus*, *Pseudomonas*, *Ralstonia*, *Rickettsia*, *Salmonella*, *Serratia*, *Shigella*, *Staphylococcus*, *Streptococcus*, *Treponema*, *Vibrio*, *Yersinia*.

## Results and Discussions

### General overview

**Fig 2:**
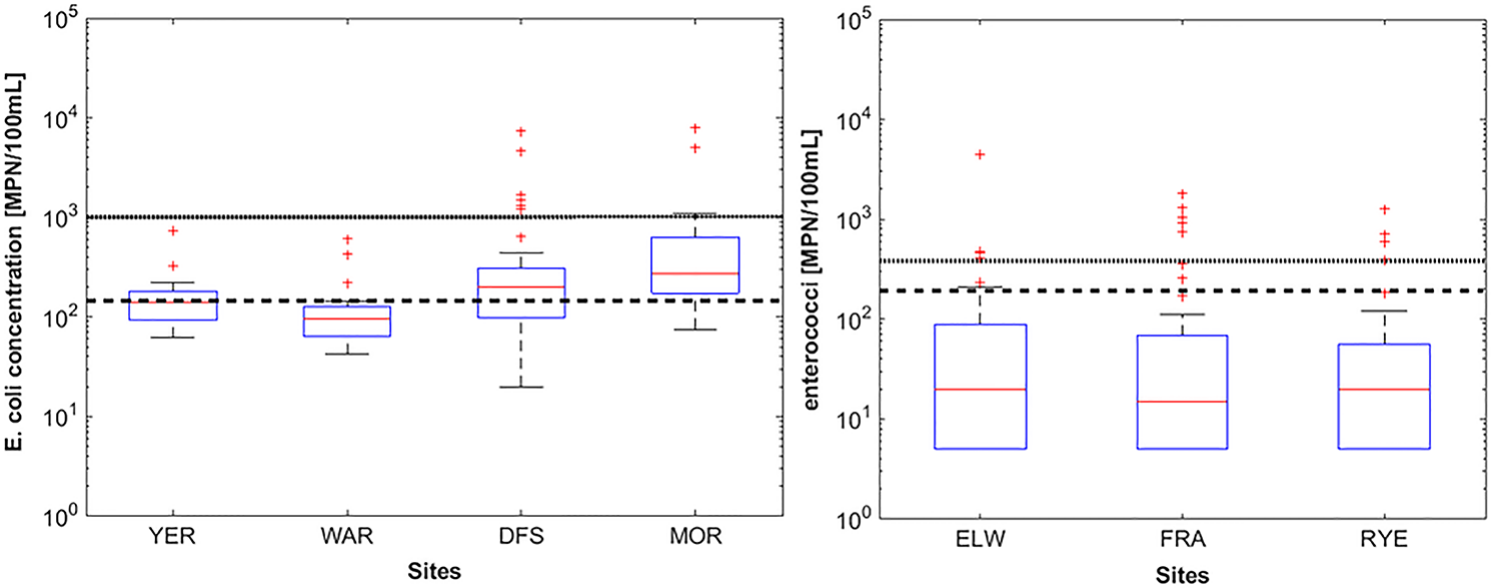
*E*. *coli* concentration at the four upstream sites (left) and enterococci concentration recorded at the three beach sites (right). The dashed and plain lines represents the SEPP thresholds for waters used for primary and secondary contact recreation in the Yarra River, respectively (*E*. *coli* = 150 MPN/100 mL and 1000 MPN/100 mL). For the marine water (right) the plain line represents the two thresholds mentioned in the SEPP for Water of Port Phillip Bay that are commonly used for long-term water quality assessment.

All three beach sites showed comparable results, with median values for IDEXX enterococci concentrations falling between 15 and 20 MPN/100 mL (Table 4, Fig 2). All sites were compliant with the two SEPP requirements for in-shore segments of Port Phillip Bay [1], having overall geometric mean below 200 MPN/100 mL and 80th-percentile level below 400 MPN/100 mL. As for day-by-day concentration, the lowest levels were observed at ELW, where just 13% of enterococci samples exceeded the 200 MPN/ 100 mL threshold. ELW also gained the highest assessment category out of the four sites, according to the NHMRC Guidelines for Managing Risks in Recreational Water Quality [22], which establish a gastrointestinal risk (GI risk) of 5%–10% for this category. All other sites were ranked in the lowest microbial assessment category D (>10% GI risk) (Table 4). Over 3 months of monitoring, the enterococci concentration at ELW, FRA, and RYE exceeded the SEPP indicator of 400 MPN/100 mL 10% of the time on average. Similarly WAR, which is part of the Yarra Watch program, complied over the testing period with both the primary and secondary contact recreation values given in SEPP for the Yarra Catchment [2], with an overall *E*. *coli* median value of 131 MPN/100 mL. MOR and DFS were the only Yarra sites that did not meet the SEPP standard for long-term primary-contact recreation.

**Table 4:** Summary statistics for E. coli and enterococci concentrations estimated using IDEXX Colilert and Enterolert, Median [5th; 95th]. n is the number of samples, with [>dl] being the number of samples greater than the detection limit indicated. The water quality assessment category was estimated using NHMRC Guidelines for Managing Risks in Recreational Water Quality [22]. The % exceedance and the long-term water quality assessment were based on SEPP values [1, 2] for fresh and marine water.

	Summary statistics					Comparison to guidelines [% exceedance]			
					NHMRC	SEPP for Yarra Catchment		SEPP for Port Phillip Bay	
					water-quality	[*E*.* coli* orgs/100 mL]		[enterococci orgs/100 mL]	
Site	*E*.* coli* [MPN/100 mL]	n [>dl]	enterococci [MPN/100 mL]	n [>dl]	assessment category	Primary [>150]	Secondary [>1000]	[>200]	[>400]
YER	141	16 [16]	**-**	**-**	**-**	50	0	**-**	**-**
	[79, 429]								
WAR	131	23	**-**	**-**	**-**	13	0	**-**	**-**
	[53, 409]								
DFS	201	34 [34]	52	13 [13]	D	59	18	**-**	**-**
	[60, 2760]		[20, 508]						
MOR	270	34 [34]	286	13 [13]	D	79	15	**-**	**-**
	[98, 6020]		[136, 1337]						
ELW	-	-	20	47 [31]	C	-	-	13^1^	9^2^
			[<10, 446]						
FRA	-	-	15	48 [35]	D	-	-	15^1^	10^2^
			[<10, 995]						
RYE	-	-	20	31 [22]	D	-	-	13^1^	10^2^
			[<10, 656]						

^1^ Geometric mean over the monitoring period was <200 orgs/100 mL, the first long-term objective for bacteriological indicators in the SEPP for Port Phillip Bay.

^2^ 80th-percentile over the testing period was <400 orgs/100 mL, the second long-term objective for bacteriological indicators in the SEPP for Port Phillip Bay.

**Fig 3:**
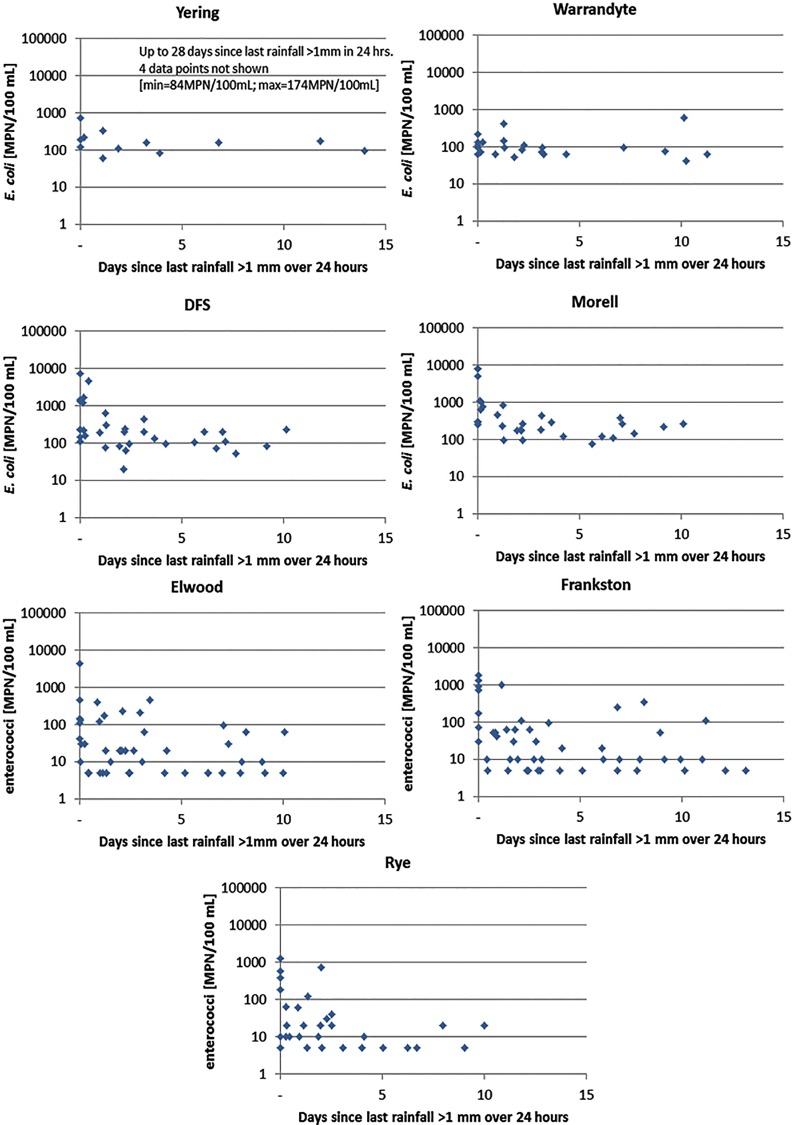
*E*. *coli* (at YER, WAR, DFS and MOR) and enterococci (at ELW, FRA, and RYE): concentrations vs time since last rainfall of >1 mm over 24 hours.

Rainfall is commonly a key explanatory factor for the within-site variability in FIOs observed in Fig 3. Rainfall-runoff processes provide the energy required to release, mobilise, and transport microbes out of surface and subsurface faecal reservoirs from urban and rural catchments into receiving water bodies [41, 42]. During rainfall events greater than 1 in 5 average recurrence interval, those processes can include human sewage discharges derived from sanitary sewer overflows, through emergency relief structures [41]. Accordingly, many jurisdictions use rainfall as a predictor variable when deciding to close recreational waters or to provide warnings to users [43]. Fig 3 shows *E*. *coli* and enterococci concentrations at each site against the antecedent dry weather period (that is, time since last rainfall-runoff event). The pattern observed at upstream sites (YER, DFS, and MOR) shows high or variable *E*. *coli* concentrations during and just after rainfall events, before returning to more consistent background levels after around 48–72 hours without rainfall. This pattern was not so obvious at the beach sites (ELW. FRA, RYE), which display a wider variation in enterococci concentrations even after a significant period of dry weather. Other inputs are therefore driving enterococci levels at those sites, confirming that rainfall is insufficient as a sole predictor variable for communicating to the public. This may suggest that more regular sampling is required for some systems, since FIOs can spike during dry weather. Literature and industry experience suggests that these spikes have several causes, including the intrusion of contaminated groundwater [44], dry weather sewage overflows [45], re-suspension of bed sediments that harbour FIOs [46], wildlife [47] or bather shedding [48].

### Inter-laboratory comparison

**Fig 4:**
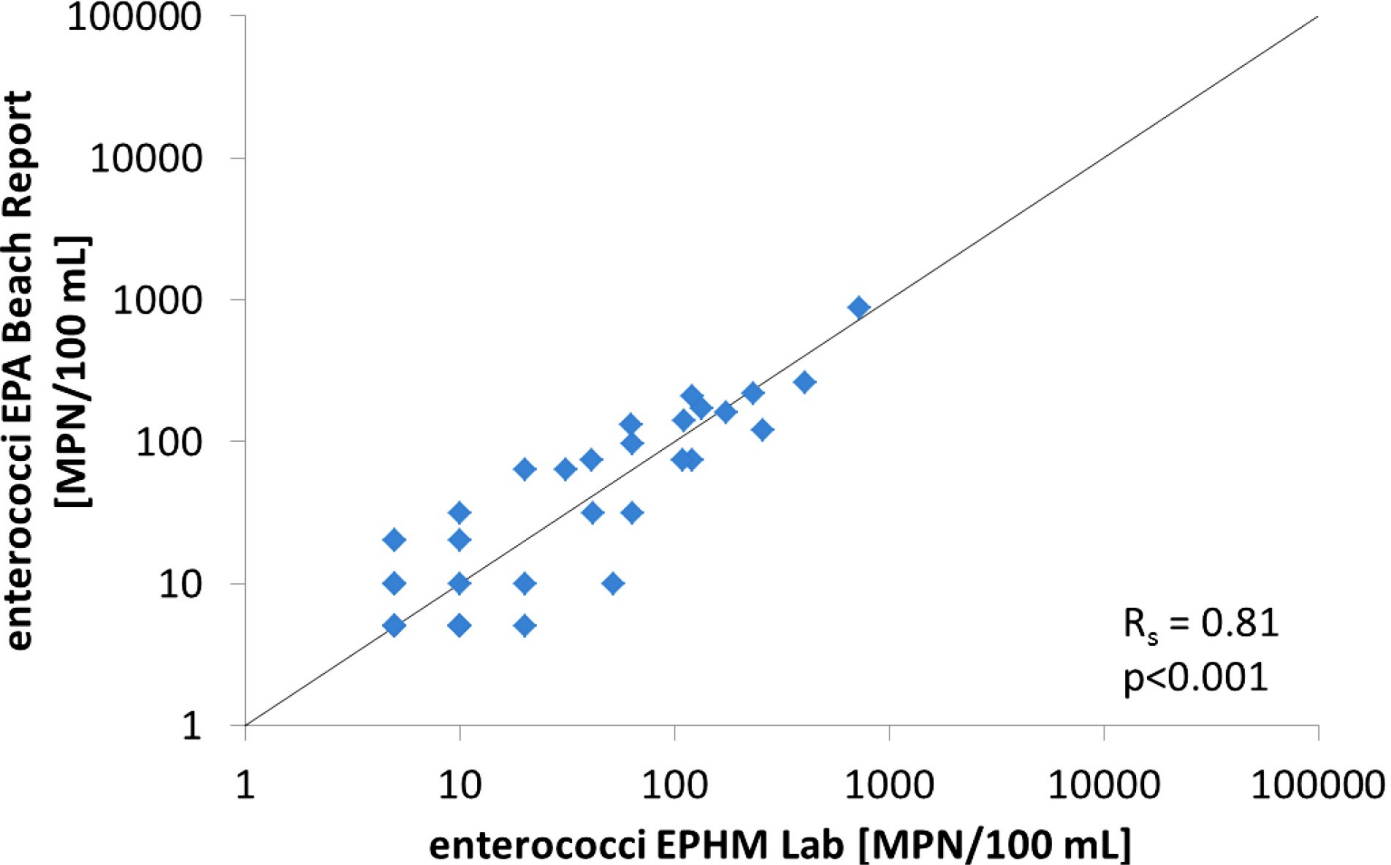
Enterococci concentration measured in the EPHM laboratory vs concentration reported on the EPA Beach Report website. Both results are from the IDEXX Enterolert method.

Results from the two laboratories were strongly correlated (R_s_ = 0.81, p<0.001) and follow the 1:1 relationship (Fig 4). The biggest variations were observed for the lower concentrations, especially those close to the lower detection limit of the Enterolert method (<10 MPN/100 mL).

### Novel methods for detection of *E*. *coli* and enterococci

#### TECTA™ vs IDEXX

**Fig 5:**
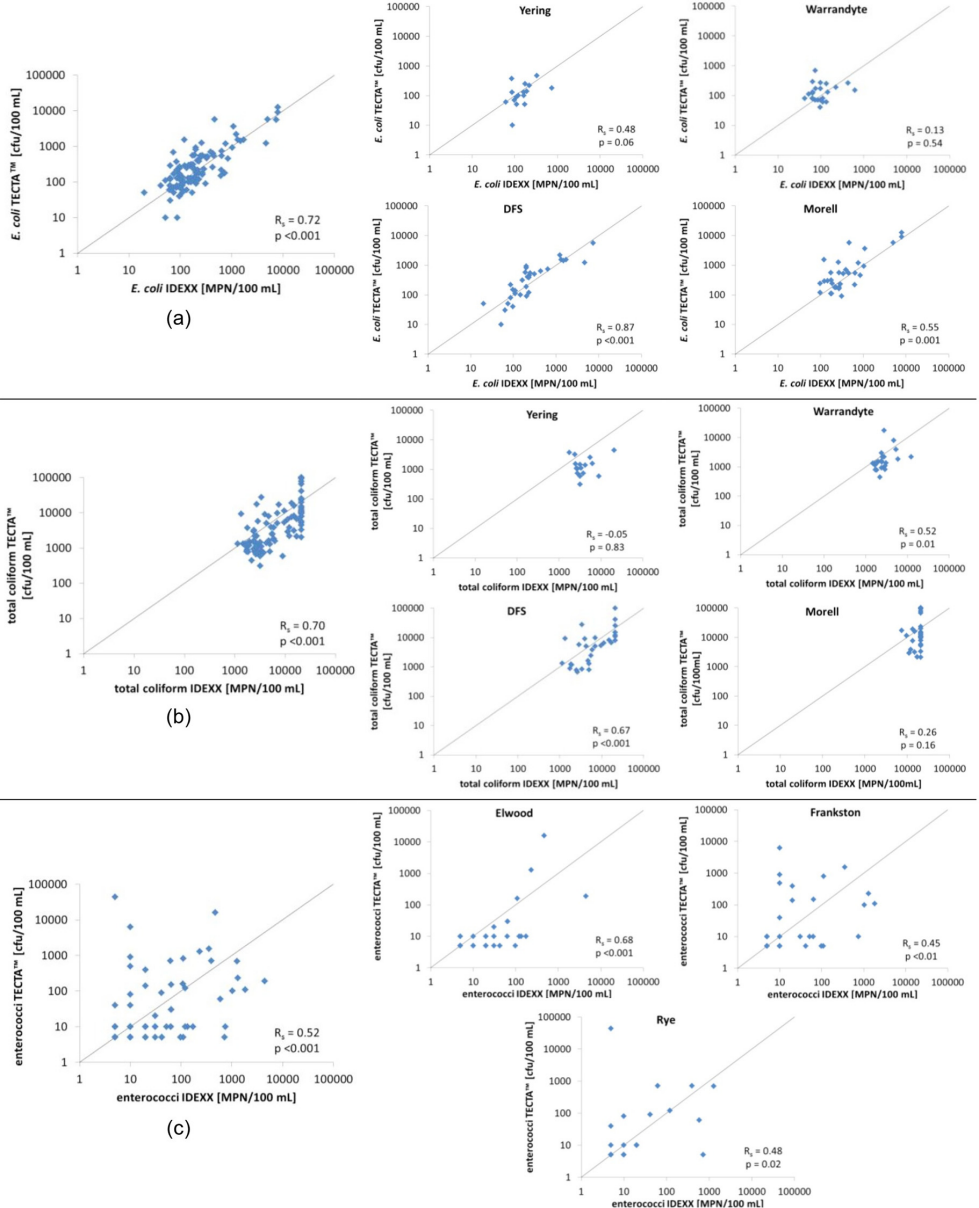
Method comparison: overall and site-specific. **(a) *E*. *coli* TECTA™ vs IDEXX Colilert; (b) total coliform TECTA™ vs IDEXX Colilert; and (c) Enterococci TECTA™ vs IDEXX Enterolert**. In each plot, the solid lines represent the 1:1 relationship.

These methods were significantly correlated for both *E*. *coli* and total coliform concentrations (*E*. *coli* R_s_ = 0.72, p<0.001; total coliforms R_s_ = 0.70, p<0.001; see Fig 5A and 5B). The lower Spearman Rank coefficient for total coliforms could be partly attributed to the IDEXX method’s upper detection limit of 20820 MPN/100 mL, while the TECTA™ method never reached its upper detection limit (Fig 5B). The results follow the 1:1 relationship (Fig 5A and 5B), showing that neither method consistently over- or under-estimated the sample concentrations with respect to one another. Although statistically significant (p<0.001), the correlations between the IDEXX and TECTA™ methods for enterococci concentrations were weaker (R_S_ = 0.51; see Fig 5C). The smaller R_s_ for enterococci was expected; indeed, TECTA™ kindly supplied their enterococci testing reagents before their final testing, and so this was considered purely as an experiment designed to help optimise their enterococci reagent.

#### qPCR vs IDEXX

**Fig 6:**
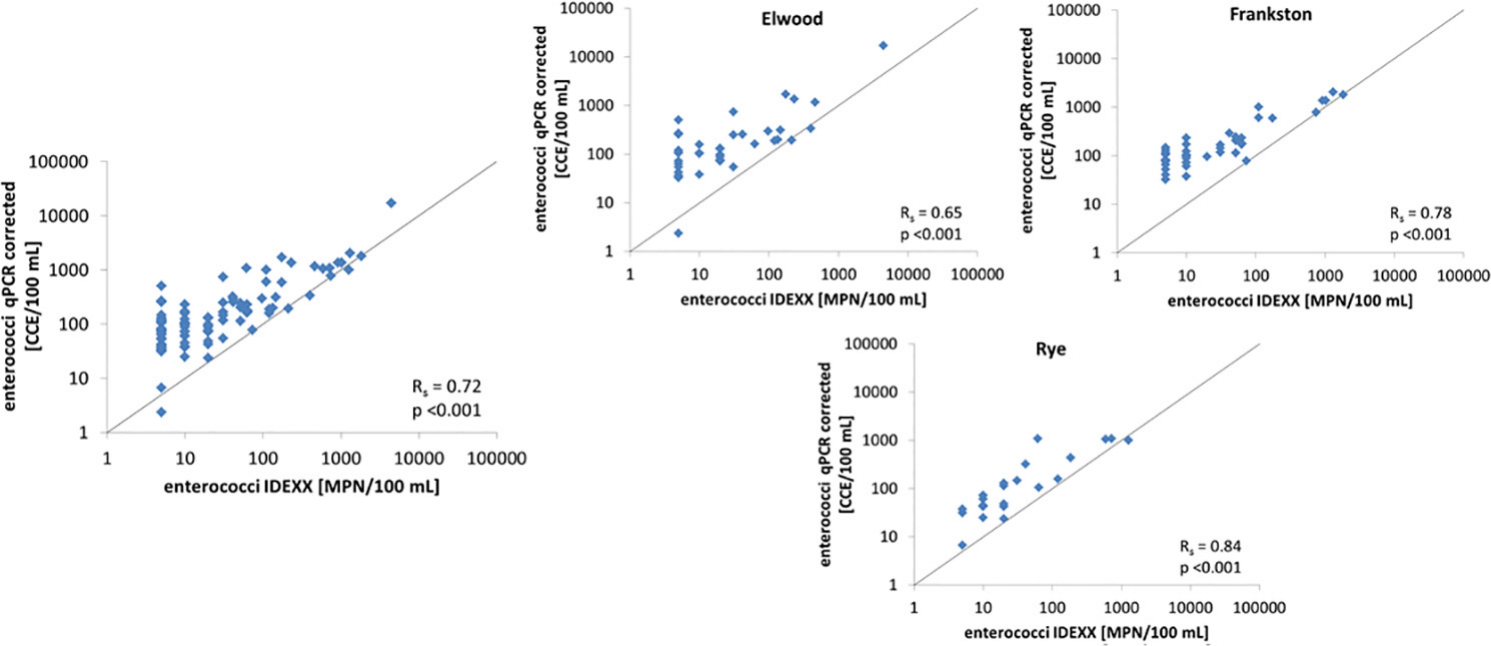
Method comparison: overall and site-specific, enterococci US EPA Method 1611 qPCR vs IDEXX Enterolert. In each plot, solid lines represent the 1:1 relationship.

IDEXX Enterolert and US EPA Method 1611 qPCR showed significant correlation (R_s_ = 0.72, p<0.001; see Fig 6). Overall, Method 1611 results nearly always overestimated the enterococci concentrations in comparison to the IDEXX method. This is not surprising, especially since 1) molecular methods can recover data from dead cells, and 2) only a fixed factor was used to correct these molecular results to cell concentrations (see Methods), yet it is entirely plausible that this correction will vary due to a number of factors (including turbidity of the sample, level of humics, salinity, etc.; we tested for these variables, but there were insufficient trends to warrant a variable correction factor).

#### IDEXX vs NGS

**Fig 7:**
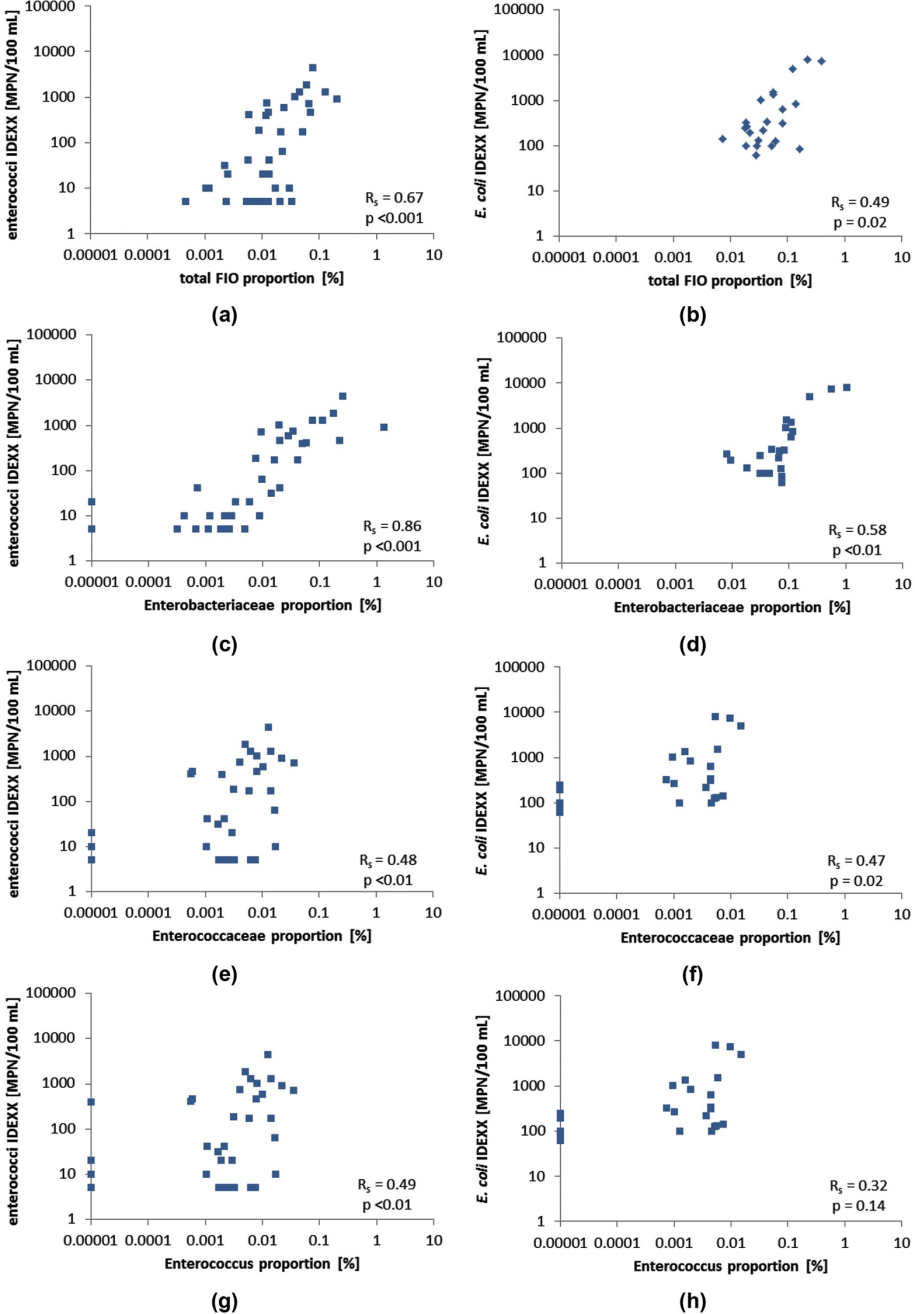
Comparison between IDEXX enterococci concentration recorded at ELW, FRA, and RYE, and IDEXX *E*. *coli* concentrations observed at MOR, DFS, WAR, and YER vs proportion of total FIOs (a and b), Enterobacteriaceae family (c and d), Enterococcaceae family (e and f), and Enterococcus genus (g and h) as measured by the NGS method.

*E*. *coli* and enterococci are commonly used faecal indicators for recreational guidelines, but they have been highly criticised for inaccuracy, inability to identify recent faecal contaminations [49], and capacity to survive in the environment [50]. With recent developments and research on community profiling, there is hope of using multiple lines of evidence to improve our understanding of hazards [28]. The first step toward these advanced methods is to verify whether they show trends similar to traditional markers of faecal contamination. Fig 7 presents a comparison between the traditional culture method of *E*. *coli* and enterococci vs chosen indicators detected by sequencing. Both *E*. *coli* and enterococci culture concentrations were significantly correlated to the total proportion of sequences from each sample belonging to our defined FIO group (R_s_ = 0.48; p<0.02 for *E*. *coli* and R_s_ = 0.67; p<0.001 for enterococci; Fig 7A and 7B). This result confirms the alignment of these methods in identifying faecal contamination, even though for each method the processing, analysis, and post-analysis were done completely independently (that is, dilution and culture vs filtering, extraction, amplification, and sequencing).

Culture-based enterococci concentrations were also well correlated with the proportion of sequences belonging to the Enterobacteriaceae family (R_s_ = 0.86, p<0.001; Fig 7C) and the Enterococcaceae family (R_s_ = 0.48, p<0.01; Fig 7E). Significant relationships were also found for *E*. *coli* and these two families (R_s_ = 0.58, p<0.01; Fig 7D and R_s_ = 0.47, p<0.01; Fig 7F respectively). Interestingly, the relationship between enterococci and Enterobacteriaceae had a greater significance than with Enterococcaceae despite the fact that enterococci are members of this family. The relationship may be explained by the fact that all three methods simply responded to increased faecal contamination (as all of them detect many bacteria found in the faeces of warm-blooded animals), and that they all have inherent uncertainties in their measurements resulting in the observed correlations.

Comparisons between culture-based methods and more specific NGS results (for example, at genus or species level) either were not possible (because *Escherichia* could not be defined to genus level due to sequence similarities to *Shigella*) or yielded poorer results (IDEXX enterococci vs *Enterococcus*; R_s_ = 0.49 p<0.01; Fig 7G), possibly reflecting the uncertainties and limitations of culture [51, 52] and NGS methods [53]. It is important to note that for NGS, these genera and species are usually found within the rare biosphere of complex environmental samples (that is, organisms present at <0.01% of the total bacterial community [54]). Sequences associated with these organisms are therefore often “removed” during post-processing quality control, resulting in an underestimation by NGS of their actual abundance. The application of family-level data enables the reads from several “rare” faecal organisms to be combined, strengthening the observed correlations with culture-derived data.

This study showed promising results for family-level comparisons, suggesting that NGS could be used not only to measure overall faecal-contamination levels in water systems (by using Enterobacteriaceae or our defined FIO group), but also to extract more specific information regarding genera and species of pathogenic bacteria likely to be present. Indeed, sequences belonging to *Clostridium*, *Aeromonas*, *Legionella*, *Salmonella Serratia* and *Vibrio* were detected in the samples. Sequence specific analysis was conducted and demonstrated that species level information could be obtained for some genera. For example, sequences specifically belonging to the pathogens *Aeromonas hydrophila* and *C*. *perfringens* were identified; both of which are faecal derived bacteria (data not shown). However, further analysis is required, through the amplification of genes specific to these species, to conclusively demonstrate the presence of these pathogens. Of the faecal pathogens relevant to recreational guidelines and microbial risk assessment, *Campylobacter* spp. sequences were measured in two fresh water samples (DFS and MOR) and more interestingly in three beach water samples (once at ELW, 3 December 2014; twice at RYE, 8 and 13 January 2015). These three occurrences correspond to days with rainfall in the preceding 24–48 hours which follows what was found in Henry et al. (2015) [55] where *Campylobacter spp*. concentrations in the water column had a positive relationship with antecedent rainfalls

### Comparison time, cost, and accuracy

Table 5 summarises the different processing and turnaround times, as well as the costs and accuracies of the methods tested. The mean detection time for samples above the detection limit of the instrument by the TECTA™ method was 13 hours for total coliforms, 12 hours for *E*. *coli* and 12 hours for enterococci –against the 24 hours necessary for both IDEXX methods. These TECTA™ results are comparable to those described in [10], where the mean detection time was 7 hours but for much higher average concentrations (which are known to provide faster reporting times for TECTA™). For the qPCR method, results were made available on average 6 hours from when samples were delivered (including processing and calculation times). Providing results on the evening of the sample collection (qPCR) or the following morning (TECTA™) translated to significant time savings for decision makers in providing warnings to the community. Costs of consumables for the TECTA™ tests were roughly equivalent to those for IDEXX, while those for the qPCR method were higher by a factor of 3.6 (LifeTech consumables were used, increasing analysis costs but reducing operator time). Operator times for the IDEXX system were roughly 7 min per sample (5 min on sampling day and 2 min the following day). This was reduced for the TECTA™ system, which requires 5 min on the sampling day only (no time the following day, because reporting is automatic; reports are emailed to the operator or end-user). Operator times for the qPCR method were estimated at around 20 min per sample. In terms of operator skills, the qPCR method requires specifically trained personnel while the TECTA™ system was easy to use and did not require extensive training. NGS processing and analysis times were significantly greater than for the other methods, while costs were about 30 times that of the IDEXX methods. Future technological advances may reduce the costs and times required for this method. In fact, the authors have begun the development of another NGS method, relying on short read lengths (50 bp vs 600 bp), thereby reducing costs by around 20% or 40% and turnaround times to less than 15 hours.

**Table 5:** Costs and processing times of the different methods, based on 2014–2015 summer monitoring. All values were estimated on the assumption that a batch of 15 samples are analysed. Consumable costs were estimated as a ratio in comparison to IDEXX consumables.

Method	Organism	Pre-processing	Post-result	Average	Cost	R_s_, p
		operator time	operator time	turnaround time		
		[min/sample]	[min/sample]	[hours]	$/$IDEXX	(vs. IDEXX)
IDEXX	Total coliform	5	2	24	1.0	–
	*E*.* coli*	5	2	24	1.0	–
	enterococci	5	2	24	1.0	–
TECTA™	Total coliform	5	0	13	0.8	R_s_ = 0.70; p<0.001
	*E*.* coli*	5	0	12	0.8	R_s_ = 0.72; p<0.001
	enterococci	6	0	12	0.7	R_s_ = 0.52; p<0.001
Method 1611	enterococci	20	5	6	3.3	R_s_ = 0.72; p<0.001
NGS	-	60^1^	30^2^	60^3^	30.0	–

^1^ Processing time for NGS includes filtration and DNA extraction.

^2^ Analysis time includes the running of scripts to pull out FIO and pathogen lists.

^3^ Average turnaround time includes all required steps to produce NGS data, including: filtration, DNA extraction, PCR amplification, Illumina MiSeq v2 sequencing, and closed OTU picking.

## Conclusions

This study is the first to compare four methods used to identify microbial concentration in recreational waters. Good correlations were observed between the TECTA™ and the IDEXX methods for *E*. *coli* and total coliforms, and promising results for the TECTA™ enterococci method. Further adjustments of the TECTA™ protocol for the benchtop instrument and the internal enterococci reagents are currently under development. The system should allow next-morning reporting to authorities, allowing them to act faster than with other culture-based methods.

Good correlations were also found between the IDEXX culture-based method and the US EPA Method 1611 for qPCR detection of enterococci in recreational waters. While this method is more expensive and requires specifically trained personnel, it also offers the potential of reporting microbial water quality of the beaches on the same day, allowing water authorities to respond and issue notifications quickly. Next-Generation Sequencing (NGS) was also promising in use, with good correlations between some family-level bacteria and traditional culture-based methods.

These new sequencing tools provide opportunities to explore more comprehensively the insidious hazards posed by pathogenic contamination of our recreational waters –a result impossible to achieve using culture-based methods.

## Supporting Information

S1 Appendix NGS analylis–QIIME scripts. (DOCX)

S2 Appendix NGS analysis—Detailed species list included in community profile search. (DOCX)
