# Supplementary material for: Evaluation of Techniques for Measuring Microbial Hazards in Bathing Waters: A Comparative Study
Source: PLoS One. 2016 May 23;11(5):e0155848. doi: 10.1371/journal.pone.0155848 (PMC4877094; doi:10.1371/journal.pone.0155848)
Supplement: S1 Appendix — (DOCX) [file pone.0155848.s001.docx]

# S1 Appendix – NGS analysis - Scripts Run: Separation of specific genus.

## Run 5.

filter_taxa_from_otu_table.py -i /data/rebekah_data/EPA/Bioms/unaltered_bioms/meta5_otu_table_closed.biom -o /data/rebekah_data/EPA/filtered_taxa/run5/meta5_otu_table_filtered_genus.biom -p g__Bacteroides,g__Bifidobacterium,g__Citrobacter,g__Enterobacter,g__Campylobacter,g__Enterococcus,g__Escherichia,g__Klebsiella,g__Salmonella,s__perfringens,s__aeruginosa,s__coli,s__jejuni,s__influenzae,s__parainfluenzae,s__aureus,s__sonnei,s__borgpetersenii,s__interrogans

summarize_taxa.py -i /data/rebekah_data/EPA/filtered_taxa/run5/meta5_otu_table_filtered_genus.biom -o /data/rebekah_data/EPA/filtered_taxa/run5/ -L 7

biom summarize-table -i /data/rebekah_data/EPA/filtered_taxa/run5/meta5_otu_table_filtered_genus.biom -o /data/rebekah_data/EPA/filtered_taxa/run5/meta5_otu_table_filtered_genus_summary.txt

biom summarize-table -i /data/rebekah_data/EPA/Bioms/unaltered_bioms/meta5_otu_table_closed.biom -o /data/rebekah_data/EPA/Bioms/unaltered_bioms/meta5_otu_table_summary.txt

- Enterobacteriaceae

filter_taxa_from_otu_table.py -i /data/rebekah_data/EPA/Bioms/unaltered_bioms/meta5_otu_table_closed.biom -o /data/rebekah_data/EPA/filtered_taxa/run5/meta5_otu_table_filtered_enterobacteriaceae.biom -p f__Enterobacteriaceae

summarize_taxa.py -i /data/rebekah_data/EPA/filtered_taxa/run5/meta5_otu_table_filtered_enterobacteriaceae.biom -o /data/rebekah_data/EPA/filtered_taxa/run5/ -L 7

biom summarize-table -i /data/rebekah_data/EPA/filtered_taxa/run5/meta5_otu_table_filtered_enterobacteriaceae.biom -o /data/rebekah_data/EPA/filtered_taxa/run5/meta5_otu_table_filtered_enterobacteriaceae_summary.txt

- FIOs

filter_taxa_from_otu_table.py -i /data/rebekah_data/EPA/Bioms/unaltered_bioms/meta5_otu_table_closed.biom -o /data/rebekah_data/EPA/filtered_taxa/run5/meta5_otu_table_fios.biom -p g__Aeromonas,g__Bacteroides,g__Bifidobacterium,g__Citrobacter,g__Enterobacter,g__Eubacterium,g__Methanobrevibacter,g__Aeromonas,g__Campylobacter,g__Enterococcus,g__Escherichia,g__Klebsiella,g__Moraxella,g__Proteus,g__Salmonella,s__perfringens,s__aeruginosa,s__coli,s__jejuni

filter_taxa_from_otu_table.py -i /data/rebekah_data/EPA/Bioms/unaltered_bioms/meta5_otu_table_closed.biom -o /data/rebekah_data/EPA/filtered_taxa/run5/meta5_otu_table_pathogens.biom -p s__phagocytophilum,s__anthracis,s__cereus,s__quintana,s__henselae,s__pertussis,s__burgdorferi,s__liberibacter,s__meningosepticum,s__diphtheriae,s__xerosis,s__amycolatum,s__ducreyi,s__aegyptius,s__pylori,s__meningitidis,s__gonorrhoeae,s__sennetsu,s__tsutsugamushi,s__solanacearum,s__rickettsii,s__conorii,s__typhi,s__prowazekii,s__influenzae,s__parainfluenzae

filter_taxa_from_otu_table.py -i /data/rebekah_data/EPA/Bioms/unaltered_bioms/meta5_otu_table_closed.biom -o /data/rebekah_data/EPA/filtered_taxa/run5/meta5_otu_table_faecal_pathogens.biom -p s__enterocolitica,s__pestis,s__parahaemolyticus,s__vulnificus,s__mimicus,s__cholerae,s__natriegens,s__hollisae,s__harveyi,s__furnissii,s__fluvialis,s__fischeri,s__alginolyticus,s__pallidum,s__pyogenes,s__pneumoniae,s__aureus,s__sonnei,s__flexneri,s__boydii,s__dysenteriae,s__liquefaciens,s__rubidaea,s__odorifera,s__marcescens,s__bongori,s__paratyphi,s__typhimurium,s__typhi,s__enteritidis,s__equi,s__aeruginosa,s__mirabilis,s__acanthamoebae,s__mycoides,s__pneumoniae,s__capricolum,s__abscessus,s__scrofulaceum,s__fortuitum,s__haemophilum,s__marinum,s__xenopi,s__kansasii,s__ulcerans,s__bovis,s__avium,s__leprae,s__tuberculosis,s__catarrhalis,s__monocytogenes,s__borgpetersenii,s__interrogans,s__pneumophila,s__pneumoniae,s__tularensis,s__calcoaceticus,s__baumannii,s__hydrophila,s__melitensis,s__cepacia,s__pseudomallei,s__mallei,s__lari,s__upsaliensis,s__trachomatis,s__psittaci,s__pneumoniae,s__tetani,s__botulinum,s__difficile,s__perfringens,s__baratii,s__butyricum,s__burnetii,s__chaffeensis,s__canis,s__ewingii,s__ruminantium,s__faecium

summarize_taxa.py -i /data/rebekah_data/EPA/filtered_taxa/run5/meta5_otu_table_fios.biom -o data/rebekah_data/EPA/filtered_taxa/run5/ -L 7

summarize_taxa.py -i /data/rebekah_data/EPA/filtered_taxa/run5/meta5_otu_table_pathogens.biom -o data/rebekah_data/EPA/filtered_taxa/run5/ -L 7

summarize_taxa.py -i /data/rebekah_data/EPA/filtered_taxa/run5/meta5_otu_table_faecal_pathogens.biom -o data/rebekah_data/EPA/filtered_taxa/run5/ -L 7

## Run 6.

- FIOs

filter_taxa_from_otu_table.py -i /data/rebekah_data/EPA/Bioms/unaltered_bioms/meta6_otu_table_closed.biom -o /data/rebekah_data/EPA/filtered_taxa/run6/meta6_otu_table_filtered_genus.biom -p g__Bacteroides,g__Bifidobacteriun m,g__Citrobacter,g__Enterobacter,g__Campylobacter,g__Enterococcus,g__Escherichia,g__Klebsiella,g__Salmonella,s__perfringens,s__aeruginosa,s__coli,s__jejuni,s__influenzae,s__parainfluenzae,s__aureus,s__sonnei,s__borgpetersenii,s__interrogans

summarize_taxa.py -i /data/rebekah_data/EPA/filtered_taxa/run6/meta6_otu_table_filtered_genus.biom -o /data/rebekah_data/EPA/filtered_taxa/run6/ -L 7

biom summarize-table -i /data/rebekah_data/EPA/filtered_taxa/run6/meta6_otu_table_filtered_genus.biom -o /data/rebekah_data/EPA/filtered_taxa/run6/meta6_otu_table_filtered_genus_summary.txt

biom summarize-table -i /data/rebekah_data/EPA/Bioms/unaltered_bioms/meta6_otu_table_closed.biom -o /data/rebekah_data/EPA/Bioms/unaltered_bioms/meta6_biom summary.txt

- Enterobacteriaceae

filter_taxa_from_otu_table.py -i /data/rebekah_data/EPA/Bioms/unaltered_bioms/meta6_otu_table_closed.biom -o /data/rebekah_data/EPA/filtered_taxa/run6/meta6_otu_table_filtered_enterobacteriaceae.biom -p f__Enterobacteriaceae

summarize_taxa.py -i /data/rebekah_data/EPA/filtered_taxa/run6/meta6_otu_table_filtered_enterobacteriaceae.biom -o /data/rebekah_data/EPA/filtered_taxa/run6/ -L 7

biom summarize-table -i /data/rebekah_data/EPA/filtered_taxa/run6/meta6_otu_table_filtered_enterobacteriaceae.biom -o /data/rebekah_data/EPA/filtered_taxa/run6/meta6_otu_table_filtered_enterobacteriaceae_summary.txt

- FIOs

filter_taxa_from_otu_table.py -i /data/rebekah_data/EPA/Bioms/unaltered_bioms/meta6_otu_table_closed.biom -o /data/rebekah_data/EPA/filtered_taxa/run6/meta6_otu_table_fios.biom -p g__Aeromonas,g__Bacteroides,g__Bifidobacterium,g__Citrobacter,g__Enterobacter,g__Eubacterium,g__Methanobrevibacter,g__Aeromonas,g__Campylobacter,g__Enterococcus,g__Escherichia,g__Klebsiella,g__Moraxella,g__Proteus,g__Salmonella,s__perfringens,s__aeruginosa,s__coli,s__jejuni

filter_taxa_from_otu_table.py -i /data/rebekah_data/EPA/Bioms/unaltered_bioms/meta6_otu_table_closed.biom -o /data/rebekah_data/EPA/filtered_taxa/run6/meta6_otu_table_pathogens.biom -p s__phagocytophilum,s__anthracis,s__cereus,s__quintana,s__henselae,s__pertussis,s__burgdorferi,s__liberibacter,s__meningosepticum,s__diphtheriae,s__xerosis,s__amycolatum,s__ducreyi,s__aegyptius,s__pylori,s__meningitidis,s__gonorrhoeae,s__sennetsu,s__tsutsugamushi,s__solanacearum,s__rickettsii,s__conorii,s__typhi,s__prowazekii,s__influenzae,s__parainfluenzae

filter_taxa_from_otu_table.py -i /data/rebekah_data/EPA/Bioms/unaltered_bioms/meta6_otu_table_closed.biom -o /data/rebekah_data/EPA/filtered_taxa/run6/meta6_otu_table_faecal_pathogens.biom -p s__enterocolitica,s__pestis,s__parahaemolyticus,s__vulnificus,s__mimicus,s__cholerae,s__natriegens,s__hollisae,s__harveyi,s__furnissii,s__fluvialis,s__fischeri,s__alginolyticus,s__pallidum,s__pyogenes,s__pneumoniae,s__aureus,s__sonnei,s__flexneri,s__boydii,s__dysenteriae,s__liquefaciens,s__rubidaea,s__odorifera,s__marcescens,s__bongori,s__paratyphi,s__typhimurium,s__typhi,s__enteritidis,s__equi,s__aeruginosa,s__mirabilis,s__acanthamoebae,s__mycoides,s__pneumoniae,s__capricolum,s__abscessus,s__scrofulaceum,s__fortuitum,s__haemophilum,s__marinum,s__xenopi,s__kansasii,s__ulcerans,s__bovis,s__avium,s__leprae,s__tuberculosis,s__catarrhalis,s__monocytogenes,s__borgpetersenii,s__interrogans,s__pneumophila,s__pneumoniae,s__tularensis,s__calcoaceticus,s__baumannii,s__hydrophila,s__melitensis,s__cepacia,s__pseudomallei,s__mallei,s__lari,s__upsaliensis,s__trachomatis,s__psittaci,s__pneumoniae,s__tetani,s__botulinum,s__difficile,s__perfringens,s__baratii,s__butyricum,s__burnetii,s__chaffeensis,s__canis,s__ewingii,s__ruminantium,s__faecium

summarize_taxa.py -i /data/rebekah_data/EPA/filtered_taxa/run6/meta6_otu_table_fios.biom -o /data/rebekah_data/EPA/filtered_taxa/run6/ -L 7

summarize_taxa.py -i /data/rebekah_data/EPA/filtered_taxa/run6/meta6_otu_table_pathogens.biom -o /data/rebekah_data/EPA/filtered_taxa/run6/ -L 7

summarize_taxa.py -i /data/rebekah_data/EPA/filtered_taxa/run6/meta6_otu_table_faecal_pathogens.biom -o /data/rebekah_data/EPA/filtered_taxa/run6/ -L 7

## Run 7.

filter_taxa_from_otu_table.py -i /data/rebekah_data/EPA/Bioms/unaltered_bioms/meta7_closed.biom -o /data/rebekah_data/EPA/filtered_taxa/run7/meta7_otu_table_filtered_genus.biom -p g__Bacteroides,g__Bifidobacterium,g__Citrobacter,g__Enterobacter,g__Campylobacter,g__Enterococcus,g__Escherichia,g__Klebsiella,g__Salmonella,s__perfringens,s__aeruginosa,s__coli,s__jejuni,s__influenzae,s__parainfluenzae,s__aureus,s__sonnei,s__borgpetersenii,s__interrogans

summarize_taxa.py -i /data/rebekah_data/EPA/filtered_taxa/run7/meta7_otu_table_filtered_genus.biom -o /data/rebekah_data/EPA/filtered_taxa/run7/ -L 7

biom summarize-table -i /data/rebekah_data/EPA/filtered_taxa/run7/meta7_otu_table_filtered_genus.biom -o /data/rebekah_data/EPA/filtered_taxa/run7/meta7_otu_table_filtered_genus_summary.txt

biom summarize-table -i /data/rebekah_data/EPA/Bioms/unaltered_bioms/meta7_closed.biom -o /data/rebekah_data/EPA/Bioms/unaltered_bioms/meta7_closed_summary.txt

- Enterobacteriaceae

filter_taxa_from_otu_table.py -i /data/rebekah_data/EPA/Bioms/unaltered_bioms/meta7_closed.biom -o /data/rebekah_data/EPA/filtered_taxa/run7/meta7_otu_table_filtered_enterobacteriaceae.biom -p f__Enterobacteriaceae

summarize_taxa.py -i /data/rebekah_data/EPA/filtered_taxa/run7/meta7_otu_table_filtered_enterobacteriaceae.biom -o /data/rebekah_data/EPA/filtered_taxa/run7/ -L 7

- FIOs

filter_taxa_from_otu_table.py -i /data/rebekah_data/EPA/Bioms/unaltered_bioms/meta7_closed.biom -o /data/rebekah_data/EPA/filtered_taxa/run7/meta7_otu_table_filtered_FIOs.biom -p g__Aeromonas,g__Bacteroides,g__Bifidobacterium,g__Citrobacter,g__Enterobacter,g__Eubacterium,g__Methanobrevibacter,g__Aeromonas,g__Campylobacter,g__Enterococcus,g__Escherichia,g__Klebsiella,g__Moraxella,g__Proteus,g__Salmonella,s__perfringens,s__aeruginosa,s__coli,s__jejuni

filter_taxa_from_otu_table.py -i /data/rebekah_data/EPA/Bioms/unaltered_bioms/meta7_closed.biom -o /data/rebekah_data/EPA/filtered_taxa/run7/meta7_otu_table_filtered_pathogens.biom -p s__phagocytophilum,s__anthracis,s__cereus,s__quintana,s__henselae,s__pertussis,s__burgdorferi,s__liberibacter,s__meningosepticum,s__diphtheriae,s__xerosis,s__amycolatum,s__ducreyi,s__aegyptius,s__pylori,s__meningitidis,s__gonorrhoeae,s__sennetsu,s__tsutsugamushi,s__solanacearum,s__rickettsii,s__conorii,s__typhi,s__prowazekii,s__influenzae,s__parainfluenzae

filter_taxa_from_otu_table.py -i /data/rebekah_data/EPA/Bioms/unaltered_bioms/meta7_closed.biom -o /data/rebekah_data/EPA/filtered_taxa/run7/meta7_otu_table_filtered_faecal_pathogens.biom -p s__enterocolitica,s__pestis,s__parahaemolyticus,s__vulnificus,s__mimicus,s__cholerae,s__natriegens,s__hollisae,s__harveyi,s__furnissii,s__fluvialis,s__fischeri,s__alginolyticus,s__pallidum,s__pyogenes,s__pneumoniae,s__aureus,s__sonnei,s__flexneri,s__boydii,s__dysenteriae,s__liquefaciens,s__rubidaea,s__odorifera,s__marcescens,s__bongori,s__paratyphi,s__typhimurium,s__typhi,s__enteritidis,s__equi,s__aeruginosa,s__mirabilis,s__acanthamoebae,s__mycoides,s__pneumoniae,s__capricolum,s__abscessus,s__scrofulaceum,s__fortuitum,s__haemophilum,s__marinum,s__xenopi,s__kansasii,s__ulcerans,s__bovis,s__avium,s__leprae,s__tuberculosis,s__catarrhalis,s__monocytogenes,s__borgpetersenii,s__interrogans,s__pneumophila,s__pneumoniae,s__tularensis,s__calcoaceticus,s__baumannii,s__hydrophila,s__melitensis,s__cepacia,s__pseudomallei,s__mallei,s__lari,s__upsaliensis,s__trachomatis,s__psittaci,s__pneumoniae,s__tetani,s__botulinum,s__difficile,s__perfringens,s__baratii,s__butyricum,s__burnetii,s__chaffeensis,s__canis,s__ewingii,s__ruminantium,s__faecium

summarize_taxa.py -i /data/rebekah_data/EPA/filtered_taxa/run7/meta7_otu_table_filtered_FIOs.biom -o /data/rebekah_data/EPA/filtered_taxa/run7/ -L 7

summarize_taxa.py -i /data/rebekah_data/EPA/filtered_taxa/run7/meta7_otu_table_filtered_pathogens.biom -o /data/rebekah_data/EPA/filtered_taxa/run7/ -L 7

summarize_taxa.py -i /data/rebekah_data/EPA/filtered_taxa/run7/meta7_otu_table_filtered_faecal_pathogens.biom -o /data/rebekah_data/EPA/filtered_taxa/run7/ -L 7

biom summarize-table -i /data/rebekah_data/EPA/filtered_taxa/run6/meta6_otu_table_fios.biom -o /data/rebekah_data/EPA/filtered_taxa/run6/meta6_otu_table_fios_summary.txt

biom summarize-table -i /data/rebekah_data/EPA/filtered_taxa/run6/meta6_otu_table_faecal_pathogens.biom -o /data/rebekah_data/EPA/filtered_taxa/run6/meta6_otu_table_faecal_pathogens_summary.txt

biom summarize-table -i /data/rebekah_data/EPA/filtered_taxa/run6/meta6_otu_table_pathogens.biom -o /data/rebekah_data/EPA/filtered_taxa/run6/meta6_otu_table_pathogens_summary.txt

biom summarize-table -i /data/rebekah_data/EPA/filtered_taxa/run5/meta5_otu_table_fios.biom -o /data/rebekah_data/EPA/filtered_taxa/run5/meta5_otu_table_fios_summary.txt

biom summarize-table -i /data/rebekah_data/EPA/filtered_taxa/run5/meta5_otu_table_faecal_pathogens.biom -o /data/rebekah_data/EPA/filtered_taxa/run5/meta5_otu_table_faecal_pathogens_summary.txt

biom summarize-table -i /data/rebekah_data/EPA/filtered_taxa/run5/meta5_otu_table_pathogens.biom -o /data/rebekah_data/EPA/filtered_taxa/run5/meta5_otu_table_pathogens_summary.txt

biom summarize-table -i /data/rebekah_data/EPA/filtered_taxa/run7/meta7_otu_table_filtered_FIOs.biom -o /data/rebekah_data/EPA/filtered_taxa/run7/meta7_otu_table_filtered_FIOs_summary.txt

biom summarize-table -i /data/rebekah_data/EPA/filtered_taxa/run7/meta7_otu_table_filtered_faecal_pathogens.biom -o /data/rebekah_data/EPA/filtered_taxa/run7/meta7_otu_table_filtered_faecal_pathogens_summary.t

biom summarize-table -i /data/rebekah_data/EPA/filtered_taxa/run7/meta7_otu_table_filtered_pathogens.biom -o /data/rebekah_data/EPA/filtered_taxa/run7/meta7_otu_table_filtered_pathogens_summary.txt

## Run 8.

- Faecal pathogens

filter_taxa_from_otu_table.py -i /data/rebekah_data/EPA/Bioms/unaltered_bioms/run_8/otu_table_run8_closed.biom -o /data/rebekah_data/EPA/filtered_taxa/run8/otu_table_run8_faecal_pathogens.biom -p s__enterocolitica,s__pestis,s__parahaemolyticus,s__vulnificus,s__mimicus,s__cholerae,s__natriegens,s__hollisae,s__harveyi,s__furnissii,s__fluvialis,s__fischeri,s__alginolyticus,s__pallidum,s__pyogenes,s__pneumoniae,s__aureus,s__sonnei,s__flexneri,s__boydii,s__dysenteriae,s__liquefaciens,s__rubidaea,s__odorifera,s__marcescens,s__bongori,s__paratyphi,s__typhimurium,s__typhi,s__enteritidis,s__equi,s__aeruginosa,s__mirabilis,s__acanthamoebae,s__mycoides,s__pneumoniae,s__capricolum,s__abscessus,s__scrofulaceum,s__fortuitum,s__haemophilum,s__marinum,s__xenopi,s__kansasii,s__ulcerans,s__bovis,s__avium,s__leprae,s__tuberculosis,s__catarrhalis,s__monocytogenes,s__borgpetersenii,s__interrogans,s__pneumophila,s__pneumoniae,s__tularensis,s__calcoaceticus,s__baumannii,s__hydrophila,s__melitensis,s__cepacia,s__pseudomallei,s__mallei,s__lari,s__upsaliensis,s__trachomatis,s__psittaci,s__pneumoniae,s__tetani,s__botulinum,s__difficile,s__perfringens,s__baratii,s__butyricum,s__burnetii,s__chaffeensis,s__canis,s__ewingii,s__ruminantium,s__faecium

summarize_taxa.py -i /data/rebekah_data/EPA/filtered_taxa/run8/otu_table_run8_faecal_pathogens.biom -o /data/rebekah_data/EPA/filtered_taxa/run8/ -L 7

biom summarize-table -i /data/rebekah_data/EPA/filtered_taxa/run8/otu_table_run8_faecal_pathogens.biom -o /data/rebekah_data/EPA/filtered_taxa/run8/otu_table_run8_faecal_pathogens_summary.txt

- FIOs

filter_taxa_from_otu_table.py -i /data/rebekah_data/EPA/Bioms/unaltered_bioms/run_8/otu_table_run8_closed.biom -o /data/rebekah_data/EPA/filtered_taxa/run8/FIOs/otu_table_run8_FIOs.biom -p g__Aeromonas,g__Bacteroides,g__Bifidobacterium,g__Citrobacter,g__Enterobacter,g__Eubacterium,g__Methanobrevibacter,g__Aeromonas,g__Campylobacter,g__Enterococcus,g__Escherichia,g__Klebsiella,g__Moraxella,g__Proteus,g__Salmonella,s__perfringens,s__aeruginosa,s__coli,s__jejuni

summarize_taxa.py -i /data/rebekah_data/EPA/filtered_taxa/run8/FIOs/otu_table_run8_FIOs.biom -o /data/rebekah_data/EPA/filtered_taxa/run8/FIOs/ -L 6,7

biom summarize-table -i /data/rebekah_data/EPA/filtered_taxa/run8/FIOs/otu_table_run8_FIOs.biom -o /data/rebekah_data/EPA/filtered_taxa/run8/FIOs/otu_table_run8_FIOs_summary.txt

- Entero

filter_taxa_from_otu_table.py -i /data/rebekah_data/EPA/Bioms/unaltered_bioms/run_8/otu_table_run8_closed.biom -o /data/rebekah_data/EPA/filtered_taxa/run8/Entero/otu_table_run8_entero.biom -p f__Enterobacteriaceae

summarize_taxa.py -i /data/rebekah_data/EPA/filtered_taxa/run8/Entero/otu_table_run8_entero.biom -o /data/rebekah_data/EPA/filtered_taxa/run8/Entero/ -L 5,6,7

biom summarize-table -i /data/rebekah_data/EPA/filtered_taxa/run8/Entero/otu_table_run8_entero.biom -o /data/rebekah_data/EPA/filtered_taxa/run8/Entero/otu_table_run8_entero_summary.txt

- Pathogens

filter_taxa_from_otu_table.py -i /data/rebekah_data/EPA/Bioms/unaltered_bioms/run_8/otu_table_run8_closed.biom -o /data/rebekah_data/EPA/filtered_taxa/run8/Pathogens/otu_table_run8_pathogens.biom -p s__phagocytophilum,s__anthracis,s__cereus,s__quintana,s__henselae,s__pertussis,s__burgdorferi,s__liberibacter,s__meningosepticum,s__diphtheriae,s__xerosis,s__amycolatum,s__ducreyi,s__aegyptius,s__pylori,s__meningitidis,s__gonorrhoeae,s__sennetsu,s__tsutsugamushi,s__solanacearum,s__rickettsii,s__conorii,s__typhi,s__prowazekii,s__influenzae,s__parainfluenzae

summarize_taxa.py -i /data/rebekah_data/EPA/filtered_taxa/run8/Pathogens/otu_table_run8_pathogens.biom -o /data/rebekah_data/EPA/filtered_taxa/run8/Pathogens/ -L 6,7

biom summarize-table -i /data/rebekah_data/EPA/filtered_taxa/run8/Pathogens/otu_table_run8_pathogens.biom -o /data/rebekah_data/EPA/filtered_taxa/run8/Pathogens/otu_table_run8_pathogens_summary.txt

## All runs combined.

- Enterococcaceae

filter_taxa_from_otu_table.py -i /data/rebekah_data/EPA/Bioms/merged_bioms/all_bioms/run_1_to_8.biom -o /data/rebekah_data/EPA/filtered_taxa/all_runs/entero.biom -p f__Enterococcaceae

biom summarize-table -i /data/rebekah_data/EPA/filtered_taxa/all_runs/entero.biom -o /data/rebekah_data/EPA/filtered_taxa/all_runs/entero_summary.txt

summarize_taxa.py -i /data/rebekah_data/EPA/filtered_taxa/all_runs/entero.biom -o /data/rebekah_data/EPA/filtered_taxa/all_runs/ -L 5,6
