# Supplementary material for: Evaluation of Techniques for Measuring Microbial Hazards in Bathing Waters: A Comparative Study
Source: PLoS One. 2016 May 23;11(5):e0155848. doi: 10.1371/journal.pone.0155848 (PMC4877094; doi:10.1371/journal.pone.0155848)
Supplement: S2 Appendix — (DOCX) [file pone.0155848.s002.docx]

# S2 Appendix - NGS analysis - Detailed species list included in community profile search.

*Acinobacter calcoaceticus*, *A. baumannii*; *Aeromonas hydrophila*; *Anaplasma phagocytophilum*; *Bacillus mycoides*, *B. anthracis*, *B. cereus*; *Bartonella quintana*, *B. henselae*, *B. pertussis*; *Borrelia burgdorferi*; *Brucella melitensis*; *Burholderia cepacia*, *B. pseudomallei*, *B. mallei*; *Campylobacter lari*, *C. upsaliensis*; *Candidatus liberibacter*; *Chlamydia trachomatis*; *Chlamydophila psittaci*; *Clostridium tetani*, *C. botulinum*, *C. difficile*, *C. perfringens*, *C. baratii*, *C. butyricum*; *Corynebacterium diphtheriae*, *C. xerosis*, *C. amycolatum*; *Coxiella burnetii*; *Ehrlichia chaffeensis*, *E. canis*, *E. ewingii*, *E. ruminantium*, *E. faecium*; *Elizabeth­kingia meningosepticum*; *Francisella tularensis*; *Haemophilus ducreyi*, *H. aegyptius*, *H. influenzae*, *H. parainfluenzae*; *Helicobacter pylori*; *Klebsiella pneumoniae*; *Legionella pneumophila*; *Listeria monocytogenes*, *L. borgpetersenii*, *L. interrogans*; *Mycobacterium abscessus*, *M. scrofulaceum*, *M. fortuitum*, *M. haemophilum*, *M. marinum*, *M. xenopi*, *M. kansasii*, *M. ulcerans*, *M. avium*, *M. leprae*, *M. tuberculosis*, *M. catarrhalis*; *Mycolplasma capricolum*; *Neisseria meningitidis*, *N. gonorrhoeae*; *Neorickettsia sennetsu*; *Orientia tsutsugamushi*; *Parachlamydia acanthamoebae*; *Proteus mirabilis*; *Pseudomonas aeruginosa*; *Ralstonia solanacearum*; *Rickettsia rickettsii*, *R. conorii*, *R. prowazekii*; *Salmonella bongori*, *S. paratyphi*, *S. typhimurium*, *S. typhi*, *S. enteritidis*, *S. equi*; *Serratia liquefaciens*, *S. rubidaea*, *S. odorifera*, *S. marcescens*; *Shigella sonnei*, *S. flexneri*, *S. boydii*, *S. dysenteriae*; *Staphylococcus aureus*; *Streptococcus pyogenes*, *S. bovis*, *S. pneumoniae*; *Treponema pallidum*; *Vibrio parahaemolyticus*, *V. vulnificus*, *V. mimicus*, *V. cholerae*, *V. natriegens*, *V. hollisae*, *V. harveyi*, *V. furnissii*, *V. fluvialis*, *V. fischeri*, *V. alginolyticus*; *Yersinia enterocolitica*, *Y. pestis*.
